# Integrin α1 subunit is up-regulated in colorectal cancer

**DOI:** 10.1186/2050-7771-1-16

**Published:** 2013-03-07

**Authors:** Salah Boudjadi, Julie C Carrier, Jean-François Beaulieu

**Affiliations:** 1 Laboratory of Intestinal Physiopathology, Department of Anatomy and Cell Biology, Faculty of Medicine and Heath Sciences, Université de Sherbrooke, 3001 12th Avenue N, Sherbrooke, QC J1H 5N4, Canada; 2 Department of Medicine, Faculty of Medicine and Health Sciences, Université de Sherbrooke, 3001 12th Avenue N, Sherbrooke, QC J1H 5N4, Canada

**Keywords:** Integrin, Colorectal cancer, Tissue microarrays, Immunohistochemistry staining, QPCR

## Abstract

**Background:**

Colorectal cancer remains one of the leading causes of death from cancer in industrialized countries. Integrins are a family of heterodimeric glycoproteins involved in bidirectional cell signaling and participate in the regulation of cell shape, adhesion, migration, differentiation, gene transcription, survival and proliferation. The α1 subunit is known to be involved in RAS/ERK proliferative pathway activation and plays an important role in mammary carcinoma cell proliferation and migration. In the small intestine, α1 is present in the crypt proliferative compartment and absent in the villus, but nothing is known about its expression in the colon mucosa, or in colorectal cancer.

**Results:**

In the present study, we demonstrated that in the colon mucosa, α1 is present in the basolateral domain of the proliferative cells of the crypt, and in the surrounding myofibroblasts. We found higher levels of α1 mRNA in 86% of tumours compared to their corresponding matched margin tissues. Immunohistochemical analysis showed that α1 staining was moderate to high in 65% of tumour cells and 97% of the reactive cells surrounding the tumour cells vs 23% of normal epithelial cells.

**Conclusion:**

Our findings suggest an active role for the α1β1 integrin in colorectal cancer progression.

## Background

Colorectal cancer (CRC) is a major public health concern in industrialized countries and remains one of the leading causes of death from cancer. Its development and progression are complex events involving many factors leading to altered expression of genes and their products. Integrins are a family of cell surface αβ heterodimeric transmembrane receptors for extracellular matrix components and cell-cell interactions. These receptors play a crucial role in mediating cell signaling in response to the extracellular environment by participating in the regulation of cell shape, adhesion, migration, differentiation, gene transcription, survival and proliferation [1-3]. In this context it is not surprising to have identified integrin involvement in cancer progression. Indeed, over-expression of the αvβ3, α5β1, αvβ5 and α6β4 integrins in various cancer types and correlation with the metastatic behaviour of breast, prostate and lung cancers as well as melanomas are well documented [4]. Altered expression of integrins has also been reported in CRC [5,6]. For example, the integrin α9β1 was detected in 50% of the tumours [7] while expression of the pro-apoptotic α8β1 integrin was found to be down-regulated in CRC and the pro-proliferative variant form of the integrin α6β4 was exclusively found in CRC cells [8,9].

Other integrins could also be involved in CRC. To date, 18 α subunits and 8 β subunits are known to form 24 different non-covalently linked heterodimers [10]. Indeed, nothing is known about integrin α1β1 expression in CRC. The integrin α1 subunit is predominantly present in stromal and smooth muscle cells and fibroblasts and is generally absent from normal epithelia although it has been reported to be expressed in developing organs such as the kidney and skin [11]. In the human intestine, the α1 subunit has been found to be expressed in myofibroblasts and muscle cells as well as in a subregion of the epithelial lining, being restricted to the proliferative epithelial cell population located in the lower part of the glands [12,13]. Interestingly, this apparent correlation between α1β1 expression and the proliferative status of the cells appears to be consistent with a pro-proliferative role for α1β1 signaling involving the transmembrane caveolin-1, adaptor protein Shc and activation of the downstream RAS/ERK proliferative pathway [11,14]. These observations suggest that integrin α1β1 may be involved in CRC. In this study, as a first step to test this hypothesis, we investigated α1 integrin subunit expression in a set of colorectal adenocarcinoma specimens.

## Results and discussion

**Figure 1:**
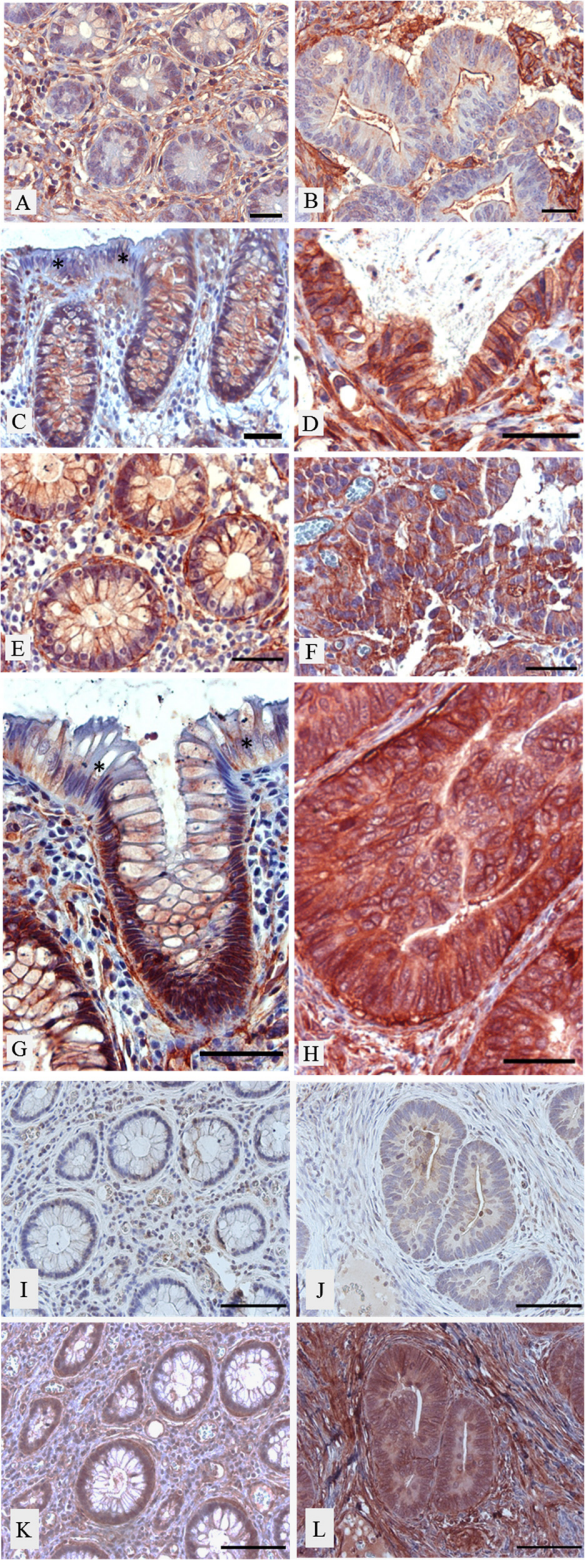
**Representative immunohistochemical images showing expression of the α1 integrin subunit in CRC (B, D, F, H, L) and corresponding matched resection margins (A, C, E, G, K).** The α1 subunit was found to be expressed at higher levels in a significant number of CRC tumours (**D**, **F**, **H**) compared to their corresponding matched normal tissues (**C**, **E**, **G**) where it was found to be predominantly expressed in the proliferative cells of the crypt and below detection levels in normal surface epithelial cells (**C**, **G**). Note that the subepithelial myofibroblasts were also stained for α1 in both normal tissues (**A**, **E**, **G**) and tumours (**B**, **D**, **F**, **H**). Scores: The margin in **A** and the tumour in **B** were both scored 0 (negative) whereas the tumour in **D** was scored 2 (strong) compared to score 0 (weak) for the matched margin **C**. The tumour in **F** was scored 1 (moderate) as was its corresponding margin in **E** (moderate). The margin in **G** was scored 0 and the matched tumour in **H** was scored 2. To validate the specificity of the primary goat anti-α1 antibody, adjacent sections of the same normal (**I**, **K**) and cancer (**J**, **L**) specimens were stained using 5 μg/ml of non-immune IgG (**I**, **J**) or anti-α1 IgG (**K**, **L**) as primary antibody. Scale bars = 50 μm.

**Figure 2:**
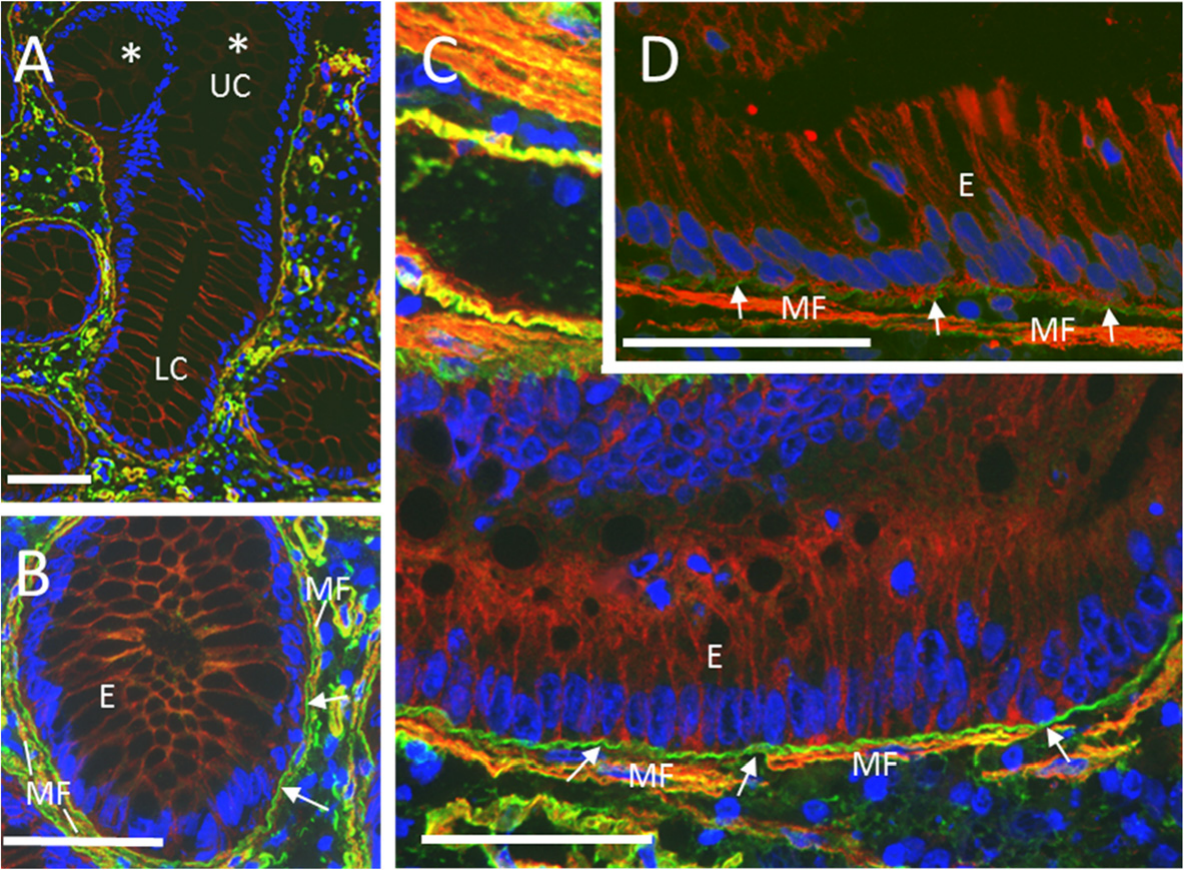
**Representative indirect immunofluorescence images showing double-stained sections for the detection of the α1 integrin subunit and laminin in a normal colon mucosa (A and B) and a moderately differentiated colon adenocarcinoma specimen (C, D).** The α1 subunit (red signal) was observed at the basolateral domain of normal epithelial cells of the lower crypt compartment (LC in panel **A** and E in panel **B**) compared to weak signal in the upper compartment (UC, panel **A**). In CRC, α1 was also localized at the basolateral domain of tumour epithelial cells (E) as well as in the adjacent subepithelial myofibroblasts (MF) (Panels **C**, **D**). Basement membrane (arrows) located at the interface between the two tissues was stained with an anti-laminin antibody (green staining). Nuclei were stained with DAPI (blue). Scale bar = 50 μm.

In the digestive system, α1 integrin subunit expression in epithelia has only been reported in the small intestine and found to be confined to the lower crypts which contain the progenitor cells [12,13]. The integrin α1 subunit’s β partner, β1, has been observed throughout the crypt-villus axis [12]. In the present study we confirmed using two distinct antibodies that, as seen in the small intestine, the α1 subunit was confined to crypt cells and was below the detection level in the differentiated epithelial cells of both the upper gland and surface epithelium of the normal colon (see * in Figure 1C, G and Figure 2A). In the lower half of the glands α1 expression was typically restricted to the basolateral domain of the epithelial cells and was also strongly expressed in the myofibroblasts [15] surrounding the crypts (Figure 1E, G, K and Figure 2B). Expression of α1 in crypt cells suggests a possible role in cell proliferation, as has been reported in mouse breast cancer [16]. As in normal epithelial cells, the α1 integrin subunit in cancers was localized at the basolateral domain of the tumour cells (Figure 1D, F, H). Indirect immunofluorescence on frozen tissue sections clearly confirmed the presence of the integrin α1 subunit in normal and tumour cells where an anti-laminin, a specific basement membrane marker [6], was used to delineate the epithelial staining from the strong mesenchymal signal (Figure 2).

**Figure 3:**
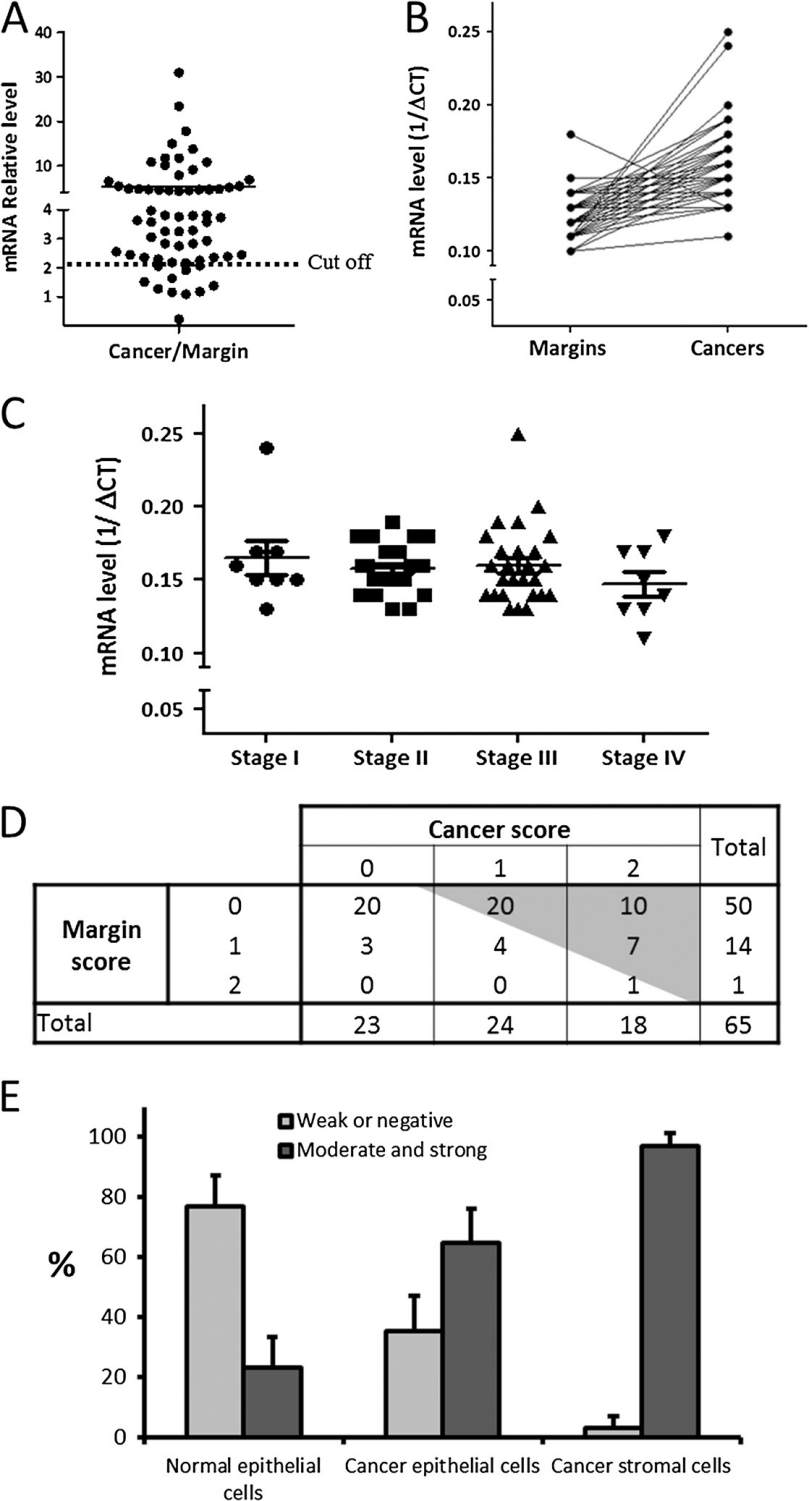
**Expression of the α1 integrin subunit in CRC samples.** (**A**) The α1 integrin subunit mRNA levels were evaluated by qPCR in 65 sets of colorectal cancers and corresponding resection matched margins. Transcript levels for α1 were found to be increased in tumours relative to their corresponding resection margins in 86% of samples (dotted line: cut off = 2). Expression of the α1 gene was calculated by the ΔΔCt method and normalized to B2M expression. (p < 0.001, One-Sample *T* Test). (**B**) To illustrate the individual tendency of paired samples, transcript levels of α1 were expressed as 1/ΔCt in each of the 65 matched margin and corresponding cancers showing the significant tendency for a higher level of expression in cancers (p < 0.001, *t*-test two-tailed). (**C**) However, α1 subunit mRNA levels did not correlate with tumour stage (stage 1; 8 patients, stage 2; 23 patients, stage 3; 26 patients and stage 4; 8 patients). (**D**) Integrin α1 expression at the protein level was analysed by immunohistochemistry on a TMA containing 65 colorectal tissues and their corresponding resection margins only considering epithelial staining. As illustrated in Figure 1, α1 immunostaining was scored as 0: negative or weak, 1: moderate or 2: strong staining. Results show that α1 staining in carcinoma vs normal epithelial cells from the matched controls was significantly higher (McNemar-Bowker’s test, p < 0.001) in 37 specimens (57%, gray area), similar in 25 specimens (38%) and lower in 3 specimens (5%). (**E**) The relative α1 integrin subunit expression was classified as negative/weak or moderate/strong. The results show that only 23% of the normal tissues displayed moderate/strong epithelial staining for α1 compared to 65% of cancer cells and 97% of the peri-tumoral stromal cells. Bars represent 95% confidence level.

Analysis of the same set of matched samples at the transcript level revealed that the mRNA levels of the α1 integrin subunit were significantly increased (from 2 up to 30 times) in 86.2% of the 65 adenocarcinomas studied when compared to their matched resection margins (Figure 3A and B). Similar increases were seen in all four stages studied (Figure 3C). However, based on the observations described above, the observed increase in α1 mRNA levels included both the tumoral and peri-tumoral tissues. Indeed, by immunohistochemistry, when considering relative protein expression only in epithelial cells, 57.0% of the tumours displayed higher expression of the α1 subunit than their matched resection control tissues (Figure 3D). In fact, relative expression analysis showed that only 23% of the control specimens displayed moderate/strong expression in the epithelium compared to 65% of the tumour specimens and 97% of the peri-tumoral stromal tissue (Figure 3E).

These results emphasize that integrin α1 subunit expression is increased in a significant proportion of both tumoral and peri-tumoral colonic tissues, a phenomenon that appears to account for the strong expression of this molecule at the transcript level. The fact that its expression is increased in 57% of the tumours relative to their matched resection margins makes the integrin α1 subunit a marker of interest in the context where the expression of other integrin subunits were found to be altered in comparable proportions such as α9 [7] and β4 [9]. Functionally, integrin α1β1 has been reported to participate in cell invasion in the hepatocarcinoma model [17] and to regulate invasion by enhancing proteinase expression in a mouse mammary carcinoma cell line [16]. In vitro adhesion studies reported that integrin α1 blocking antibodies reduced peritoneal gastric cell invasion [18] while in the colorectal cancer HT-8/S11 cell line, α1, but not α2 or β1 clustering induced the recruitment of the FAK/Src signaling complex involved in cell invasion [19]. It has also been reported that angiogenesis was reduced in integrin α1-null mice [20]. Moreover, loss of the integrin α1 subunit has been found to decrease the incidence and growth of lung epithelial tumours initiated by oncogenic Kras [21] consistent with the fact that Ras is a downstream effector of the α1β1 integrin [14] and that oncogenic changes in the Kras gene alone are not sufficient to confer a malignant phenotype [22]. Kras mutation is well known in colorectal cancer resistance to Cetuximab [23,24] but, to date, the link with α1 in CRC is not known.

On the other hand, it has recently been reported that cancer associated stromal cells have a pro-inflammatory gene signature [25] and promote cancer cell invasiveness [26,27]. Another study reported that fibroblasts could drive tumour mammary carcinoma progression by modulating biochemical forces through β1 integrin signalling [28]. The data presented herein showing up-regulation of the integrin α1 subunit in the stromal compartment of colorectal tumours may also suggest a cooperative role of the integrin α1β1 in colon cancer progression.

## Conclusions

In conclusion, the data presented in this study identified the expression and predominant localization of the α1 integrin subunit in the proliferative compartment of the normal colonic epithelium and demonstrated that α1 expression was significantly up-regulated in CRC in both tumour cells and surrounding stromal cells, suggesting a positive role for the α1β1 integrin in CRC progression.

## Methods

### Patients, tumour tissues and tissue microarrays

Primary colorectal adenocarcinomas and paired margin tissues were obtained from 65 patients undergoing surgical resection without prior neoadjuvant therapy. Tissues were obtained after patient’s written informed consent, according to a protocol approved by the Institutional Human Subject Review Board of the Centre Hospitalier Universitaire de Sherbrooke. Staging of the adenocarcinomas was according to the TNM classification of tumours. There were 8 stage 1, 23 stage 2, 26 stage 3 and 8 stage 4 specimens. For immunohistochemistry, samples were fixed with 4% paraformaldehyde in 0.1 M PBS at 4°C overnight, dehydrated in graded alcohols, and then embedded in paraffin. For cryosections, tissues were embedded as previously described [9,12]. Total RNA was extracted from tissues using the Totally RNA kit (Invitrogen, Burlington, ON) and processed according to the manufacturer’s instructions [8,9].

Tissue microarrays (TMA) were performed as previously described [29]. Briefly, 5μm thick serial sections were processed for routine hematoxylin and eosin staining, in order to hallmark the tissue region for TMA. Tissue cores with a diameter of 2 mm were removed from fixed paraffin–embedded tissue blocks using a 2 mm dermatological biopsy punch (Miltex Inc. York, PA) and arrayed in a paraffin mold which was first covered with double-sided adhesive to hold the cores in the correct position. Once all cores were deposited at the bottom of the mold, hot paraffin was poured to fill the mold and create a new block after incubation for one hour at 4°C. From the new block, sections of 5μm in thickness were made. Each section was spread on a glass slide and stored at room temperature.

### Immunohistochemistry and expression analysis

Sections (5 μm thick) cut from paraffin-embedded TMA were mounted on charged slides, deparaffinated in xylene and rehydrated in graded alcohol. Antigen retrieval was performed in 0.01M citrate buffer, pH 6, in a microwave pressure cooker for 30 minutes. Slides were cooled to room temperature before reacting with a peroxidase blocking reagent (0.3% H_2_O_2_) for 30 minutes, a streptavidin/biotin blocking reagent (Vector Laboratories Inc, Burlington, ON) for 15 min, and blocking serum [PBS 1×, 0.1% BSA (Sigma-Aldrich, Oakville, ON), 0.2% Triton X-100 (ICN Biochemicals, Aurora, OH), 0.1% donkey serum, 0.1% goat serum] for 30 minutes. Sections were incubated overnight at 4°C with anti-human integrin α1 purified polyclonal sheep IgG (5 μg/ml, AF5676, R & D Systems, Minneapolis, MN) or with equal amounts of sheep non-immune IgG (sc-2717, Santa Cruz Biotechnology, Santa Cruz, CA) as negative control, followed by incubation with an anti-sheep biotinylated secondary antibody (Vector Laboratories) for one hour at room temperature. Then, tissues were incubated with a streptavidin HRP conjugated solution (1:1000, Millipore, Billerica, MA) for one hour and the colour developed with 3,3^′^- diaminobenzidine (Vector Laboratories) in a buffered substrate solution. Slides were counterstained with light hematoxylin, dehydrated and cover-slipped. Representative images were acquired using a Leika DM-RXA microscope. Protein expression in different cell types, including epithelial tumour cells, epithelial normal cells and reactive cells, was separated into 2 groups based on staining intensity: negative/low or moderate/strong expression. Expression in tumour cells was compared to the normal epithelial cells of the respective margin and scored as 0; no or weak staining, 1; moderate, and 2; strong staining.

### Indirect immunofluorescence

To determine the α1 expression pattern, 3 μm thick sections were cut from different normal colonic mucosa and adenocarcinomas samples. First, sections were fixed 10 min in ethanol at -20°C and then washed 3 times with chilled PBS. Then, nonspecific protein-protein interactions were blocked for 30 minutes with 10% blotto followed by 2 hours incubation with the primary α1 mouse monoclonal antibodies TS27 (Endogen, Woburn, MA) diluted 1:10, and an anti-laminin rabbit antibody (Serotec, Raleigh, NC) diluted 1:1000 in 10% blotto. After three washes with ice-cold PBS, slides were incubated one hour at room temperature with AlexaFluor 488 and AlexaFluor 594 conjugated secondary antibodies directed against mouse and rabbit IgG (Molecular Probe, Burlington, ON). Slides were then stained with DAPI (4^′^,6-diamidino-2-phenylindole, 2%) and then mounted in glycerol: PBS (9:1) containing 0.1% paraphenylenediamine and observed with a Leica DM-RXA microscope. Images were acquired and composites generated with the MetaMorph Imaging System (Universal Imaging, West Chester, PA).

### Real time quantitative RT-PCR

The RNA was reverse-transcribed to cDNA with AMV reverse transcriptase (Roche, Laval, QC) following the manufacturer’s instructions. Primers used to amplify α1 were 5^′^-CATCAGGTGGGGATGGTAAG-3^′^ and 5^′^-TGGCTCAAAATTCATGGTCA-3^′^. B2M was used as housekeeping gene and primers were 5^′^-GTGCTCGCGCTACTCTCTC-3^′^ and 5^′^-GTCAACTTCAATGTCGGAT-3^′^. Quantitative PCR was performed using a MX3000P Real-Time System (Stratagene, Cedar Creek, TX). α1 gene expression was calculated by the ΔΔ*C*_T_ method and normalized to B2M expression [30].

### Statistical analysis

The One-Sample Student’s *t*-test was used to determine the statistical significance of mRNA expression analyses. For immunohistological analyses, the McNemar-Bowker’s test was used to compare scores between tumours and non-malignant samples.

## Abbreviations

CRC: Colorectal cancer; TMA: Tissue microarrays

## Competing interests

The authors declare that they have no competing interests.

## Authors’ contributions

SB carried out the experiments, participated in the analysis and interpretation of the data and has been involved in the drafting of the manuscript. JCC participated in the acquisition of the data and to the design of the study. JFB conceived of the study, participated in the interpretation of the data and in the preparation of the manuscript. All authors read and approved the final manuscript.
